# A longevity‐specific bank of induced pluripotent stem cells from centenarians and their offspring

**DOI:** 10.1111/acel.14351

**Published:** 2024-09-25

**Authors:** Todd W. Dowrey, Samuel F. Cranston, Nicholas Skvir, Yvonne Lok, Brian Gould, Bradley Petrowitz, Daniel Villar, Jidong Shan, Marianne James, Mark Dodge, Anna C. Belkina, Richard M. Giadone, Sofiya Milman, Paola Sebastiani, Thomas T. Perls, Stacy L. Andersen, George J. Murphy

**Affiliations:** ^1^ Center for Regenerative Medicine of Boston University and Boston Medical Center Boston Massachusetts USA; ^2^ Section of Hematology and Medical Oncology Boston University Chobanian & Avedisian School of Medicine Boston Massachusetts USA; ^3^ Section of Geriatrics, Department of Medicine Boston University Chobanian & Avedisian School of Medicine Boston Massachusetts USA; ^4^ Albert Einstein College of Medicine Bronx New York USA; ^5^ Flow Cytometry Core Facility Boston University Chobanian & Avedisian School of Medicine Boston Massachusetts USA; ^6^ Department of Pathology and Laboratory Medicine Boston University Chobanian & Avedisian School of Medicine Boston Massachusetts USA; ^7^ Department of Stem Cell and Regenerative Biology Harvard University Cambridge Massachusetts USA; ^8^ Institute for Clinical Research and Health Policy Studies Tufts Medical Center Boston Massachusetts USA

**Keywords:** centenarians, induced pluripotent stem cells, longevity

## Abstract

Centenarians provide a unique lens through which to study longevity, healthy aging, and resiliency. Moreover, models of *human* aging and resilience to disease that allow for the testing of potential interventions are virtually non‐existent. We obtained and characterized over 96 centenarian and offspring peripheral blood samples including those connected to functional independence data highlighting resistance to disability and cognitive impairment. Targeted methylation arrays were used in molecular aging clocks to compare and contrast differences between biological and chronological age in these specialized subjects. Isolated peripheral blood mononuclear cells (PBMCs) from 20 of these subjects were then successfully reprogrammed into high‐quality induced pluripotent stem cell (iPSC) lines which were functionally characterized for pluripotency, genomic stability, and the ability to undergo directed differentiation. The result of this work is a one‐of‐a‐kind resource for studies of human longevity and resilience that can fuel the discovery and validation of novel therapeutics for aging‐related disease.

## Graphical abstract

We collected centenarian and offspring peripheral blood samples connected to functional independence data. Isolated peripheral blood mononuclear cells (PBMCs) were used in targeted methylation arrays to fuel molecular aging clocks and establish biological age in these subjects, of which 20 were then successfully reprogrammed into high‐quality induced pluripotent stem cell (iPSC) lines. We report a one‐of‐a‐kind resource for studies of human longevity and resilience that can fuel the discovery and validation of novel therapeutics for aging‐related disease.

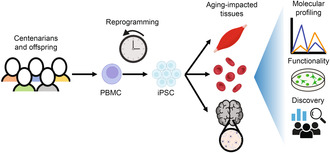

**Abbreviations**
AD: Alzheimer's disease
ADL: Activities of daily living
EL: exceptional longevity
ES: embryonic stem
IADLs: instrumental activities of daily living
ILO: Integrative Longevity Omics
iPSC: Induced pluripotent stem cell
LCCP: Longevity Consortium Centenarian Project
mLOY: mosaic loss of Y
NECS: New England Centenarian Study
NGN2: Neurogenin 2
PBMC: peripheral blood mononuclear cell

## 1 INTRODUCTION

Individuals with exceptional longevity (EL) age more successfully than the general population by extending their health span and decreasing the proportion of their lives spent with aging‐related disease, deemed the ‘compression of morbidity’ (Evert et al., 2003; Fries, 1980; Fries et al., 2011; Hitt et al., 1999). Centenarians (>100 years of age) provide a unique lens through which to study EL, healthy aging, and disease resilience and resistance (Andersen, 2020; Andersen et al., 2012). In the last decade, several studies have provided evidence that centenarians exhibit delayed onset or escape aging‐related diseases such as cancer, cardiovascular disease, and Alzheimer's disease (AD) while markedly delaying disability (Andersen et al., 2012; Evert et al., 2003; Terry et al., 2004). Although recent work has identified genetic variants associated with healthful aging, insights into how these elements promote longevity remain unclear (Lin et al., 2021). Understanding the regulatory networks that promote resistance to aging‐related disease may provide mechanistic insights into this process and inform the development of therapeutics to slow or reverse aging. Problematically, however, models of human aging, longevity, and resistance to and/or resilience against disease that allow for the functional testing of potential interventions are virtually non‐existent.

Induced pluripotent stem cells (iPSCs) faithfully capture the genetic background of the person from whom they are created and are revolutionizing pre‐clinical drug screening by exhibiting the power of precision medicine. Beginning soon after their initial discovery, iPSCs have been used to model diseases as well as screen drugs for the treatment of amyotrophic lateral sclerosis (Egawa et al., 2012), spinal muscular atrophy (Ebert et al., 2009), and other neurodegenerative (Okano & Morimoto, 2022) and muscular (Rashid et al., 2023) disorders. Notably and importantly, these model systems have been applied to and faithfully recapitulate disease pathologies associated with aging that manifest late in life, such as in AD (Cenini et al., 2020; Jorfi, D'Avanzo, Kim, & Irimia, 2018; Jorfi, D'Avanzo, Tanzi, et al., 2018; Park et al., 2018; Quinti et al., 2021) and in our own work on familial ATTR‐amyloidosis (Ghosh et al., 2023; Giadone et al., 2018, 2020; Leung et al., 2013). The versatility in these systems to produce any cell and tissue type of the body allows for interrogation of multiple, aging‐impacted tissues, many of which have been demonstrated to age at different rates (Nie et al., 2022). This potential utility of iPSCs in aging research has recently been highlighted as a platform for both longevity studies as well as drug discovery (Jothi & Kulka, 2024; Pitrez et al., 2024). EL iPSCs also provide a complement for studies performed on aging disease‐specific lines, such as those derived from patients with Progeria (Daily et al., 2023; Monnerat et al., 2022), given their potential in understanding mechanisms that embolden resiliency and resistance to accelerated aging‐related disease.

Moreover, centenarian offspring have been shown to display increased resistance to aging‐related disease (Adams et al., 2008; Andersen, 2020; Newman et al., 2011; Sebastiani et al., 2009; Terry et al., 2003, 2004) and map younger in aging rate indicators (Horvath et al., 2015; Sebastiani et al., 2021). Recent studies have included iPSC lines generated from centenarian offspring to allow for comparison to those from age‐matched controls with no familial history of EL (Aiello et al., 2019; Bucci et al., 2016).

Here, we report a novel bank of longevity‐specific peripheral blood mononuclear cells (PBMCs) and resultant iPSCs from subjects with EL including centenarians, their offspring, and offspring age‐matched controls. The result of this work is a highly characterized, one‐of‐a‐kind resource that can aid in a host of aging‐related studies. This flexible iPSC library represents a unique, permanent resource that can be harnessed by any investigator for molecular and functional analyses of resiliency, longevity, and aging. It also provides a much‐needed human platform for the discovery of novel geroprotective agents and/or the validation of findings from other data banks, tissue repositories, or models. Lastly, our ability to connect detailed phenotypic data obtained from the subjects as well as molecular and biological aging data to the created lines enables the identification of those at the extremes of both physical and cognitive functionality for study.

## 2 RESULTS

### 2.1 Identification and characterization of individuals displaying EL

PBMCs were collected from 45 centenarians (mean age at last contact/death 104.3 ± 3.4 years, 60% females), 45 offspring (mean age at last contact 76.3 ± 9.4 years, range 59–90+ years, 67% females), and 6 non‐EL offspring spouses (80.0 ± 8.4 years, 17% females) (Table 1). Among the centenarians for whom cognitive and/or functional status could be determined at age 100, 76% (16/21) were cognitively healthy and 83% (24/29) were Activities of Daily Living (ADL) independent at age 100 years. Among offspring with sufficient data to characterize current cognitive and/or functional status, 93% (25/27) were cognitively healthy and 88% (29/33) were independent in performing instrumental activities of daily living (IADLs) at the time of assessment. For investigators seeking additional protected phenotypic data on study participants, applications can be made to the ELITE portal data hub (https://eliteportal.synapse.org/) where this information is securely housed.

**TABLE 1:** Cognitive and functional status of EL subjects.

Age at draw	Sex (*N*)	Cognitively healthy at age 100 (*N*)	ADL independent at age 100 (N)	Both cognitively healthy and ADL independent at age 100 (*N*)
Centenarians				
100–104	Male (11) Female (17)	Yes (11)	Yes (20)	Yes (11)
		No (5)	No (2)	No (6)
		Unknown (12)	Unknown (6)	Unknown (11)
105–109	Male (7) Female (8)	Yes (5)	Yes (5)	Yes (4)
		No (0)	No (3)	No (3)
		Unknown (10)	Unknown (7)	Unknown (8)
110+	Male (0) Female (2)	Yes (1)	Yes (1)	Yes (1)
		No (0)	No (0)	No (0)
		Unknown (1)	Unknown (1)	Unknown (1)

Age at draw	Sex (*N*)	Cognitively healthy (*N*)	IADL independent (*N*)
Offspring			
50–59	Male (1) Female (0)	Yes (1)	Yes (0)
		No (0)	No (0)
		Unknown (0)	Unknown (1)
60–69	Male (1) Female (10)	Yes (10)	Yes (9)
		No (0)	No (0)
		Unknown (1)	Unknown (2)
70–79	Male (7) Female (11)	Yes (15)	Yes (17)
		No (1)	No (0)
		Unknown (2)	Unknown (1)
80–89	Male (4) Female (6)	Yes (4)	Yes (8)
		No (1)	No (1)
		Unknown (5)	Unknown (1)
90+	Male (2) Female (3)	Yes (2)	Yes (2)
		No (0)	No (3)
		Unknown (3)	Unknown (0)

Age at draw	Sex (*N*)	Cognitively healthy (N)	IADL independent (N)
Offspring spouses			
60–69	Male (1) Female (0)	Yes (0)	Yes (0)
		No (0)	No (0)
		Unknown (1)	Unknown (1)
70–79	Male (0) Female (0)	Yes (0)	Yes (0)
		No (0)	No (0)
		Unknown (0)	Unknown (0)
80–89	Male (4) Female (1)	Yes (2)	Yes (4)
		No (0)	No (1)
		Unknown (3)	Unknown (0)

*Note*: Demographic (age, sex), cognitive, and functional status of subjects in this bank are organized by age bracket and separated by cohort (centenarian, offspring, and offspring spouses) with number of subjects listed next to each identifier. Cognitive status is determined by clinical consensus review of neuropsychological assessment scores or cognitive screeners detailed in the methods. Functional independence status was determined by performance on the Barthel Activities of Daily Living Index (ADLs) for centenarians and instrumental activities of daily living (IADLs) for offspring and spouses.

### 2.2 Comparisons of biological versus chronological age in EL subjects

**FIGURE 1:**
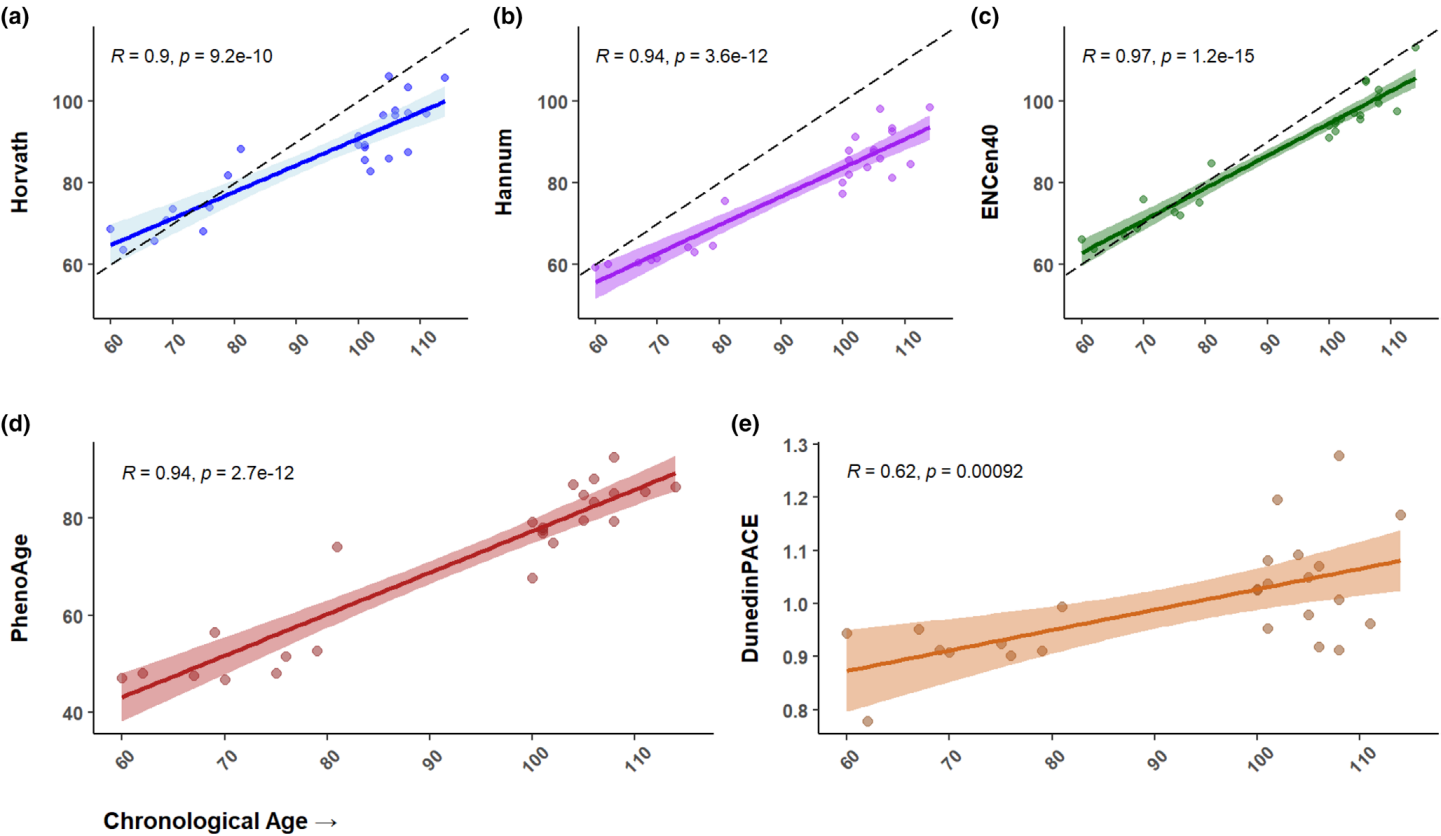
Comparisons of biological versus chronological age in EL subjects. Parallel age estimates from five well‐established models of epigenetic aging for EL PBMCs. The colored lines represent the best linear fit from each model, while the dotted black lines show a theoretical perfect linear correlation for reference, where applicable. Each data point represents an individual within our bank. The top three panels show models trained on chronological age including the original 2013 pan‐tissue Horvath (a), Hannum (b), and ENCen40+ (c) clocks. (d and e) show predictions from models (PhenoAge and DunedinPACE) trained with the additional aid of clinical and phenotypic measurements. These clocks return more novel measurements that align more closely to a putative biological age, or rate of age acceleration, as opposed to chronological age.

Methylation profiling was performed with Illumina Infinium arrays on 25 subjects in this bank which allowed for estimation of biological age through established aging clocks, including the pan‐tissue Horvath DNAmAge (Horvath, 2013; Horvath et al., 2018) clock, (Hannum et al., 2013), DunedinPACE (Belsky et al., 2022), and Phenoage (Levine et al., 2018) clocks, as well as a recently published aging clock trained on datasets that included a relatively high proportion of centenarians (ENCen40+) (Dec et al., 2023) (Figure 1). This panel of epigenetic clocks comprises a wide array of models, trained on both chronological and phenotypic measurements, and spanning multiple generations of Illumina methylation chips. To facilitate compatibility between each of these models with the newest Illumina beadchips, imputation for the small subsets of missing CpGs for each model was performed where applicable (see Section 4). Despite differences in newer generation (Illumina EPICv2) versus older generation methylation arrays, estimates and model behavior seen with our samples were largely in line with expectations. Models trained on the older Illumina 27/450k arrays saw accurate age prediction in younger individuals (Horvath median absolute error [MAE] = 2.69, Hannum MAE = 8.08 on non‐centenarian samples, with overall Pearson correlation coefficients (*R*) of 0.90 and 0.94, respectively), as well as a general under‐predictive trend in EL samples. Meanwhile, clocks measuring putative biological aging (PhenoAge, DunedinPACE) showed similarly consistent correlation. PhenoAge showed a correlation coefficient of 0.94 with chronological age, while DunedinPACE centered around an average value of 1 (representing the age acceleration of a healthy adult) with gradual increase in the age acceleration of chronologically older individuals (*R* = 0.62) as noted by the authors in the original manuscript (Belsky et al., 2022). Based on the spectrum of clocks employed, the centenarians in this bank mapped biologically younger to varying extents. Notably, the ENCen40+ clock estimated an average 6.55‐year reduction in biological age and a much higher average degree of accuracy in centenarians relative to other current models. These data reflected the relative health metrics and clinical history associated with each subject, with those displaying functional independence and higher cognitive function having the most pronounced reduction in biological age. Interestingly, EL offspring displayed more variable biological age status, with some subjects estimated to have a higher biological age as well as some estimated to have a lower biological age (Figure 1). The greater variation in the offspring may be the result of greater heterogeneity in the presence or absence of genetic signatures associated with EL. Of note, a subset of the PBMCs collected in this study were also characterized via single cell RNA sequencing and CITEseq analyses (Karagiannis et al., 2023), as well as a 40‐color flow cytometry panel to extensively examine immune cell subtypes and identify unique features in EL subjects (Figures S1 and S2). Briefly, we identified both previously reported EL immune cell repertoire changes such as a lymphoid shift to a more cytotoxic profile, as well as novel changes such as an increase in CD14^+^ monocytes and a decrease in dendritic cells. In addition to transcriptional changes common across aging, we also identified changes specific to EL immune cells enriched for protective factors and gene variants, such as *STK17A* and *S100A4* in naïve CD4^+^ T cells reported to be involved in DNA damage response and inflammatory pathways in the brain respectively (Karagiannis et al., 2023).

### 2.3 Establishment and characterization of a longevity‐specific iPSC bank

**FIGURE 2:**
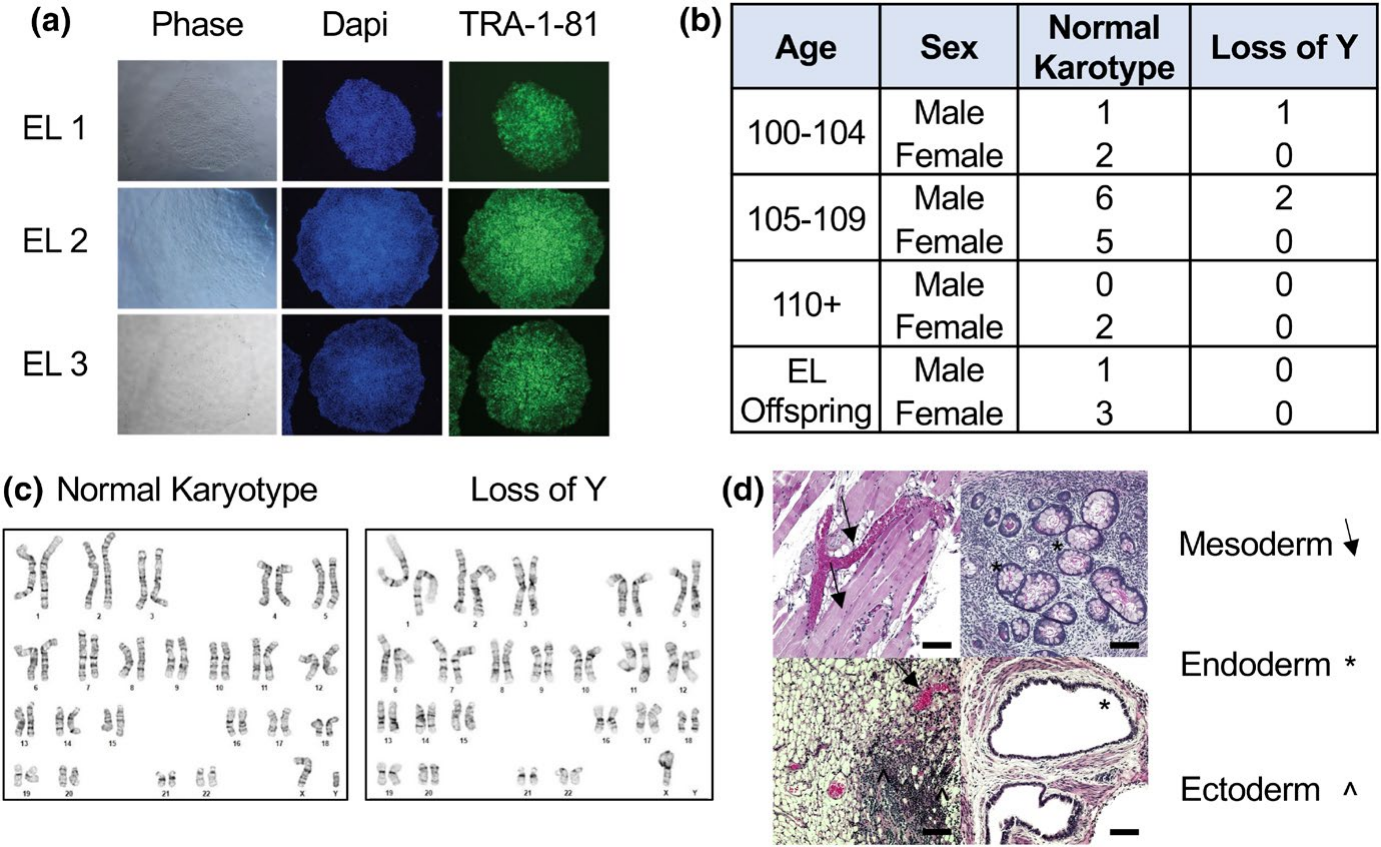
Representative characterization of EL‐specific iPSCs. (a) Representative images of iPSC lines under brightfield (left), DAPI (middle), and TRA‐1‐81 (right). Images taken at 10× magnification. (b) Table of iPSC lines generated including demographic information (age, sex) and karyotype outcome based on g‐band analyses. (c) Representative karyotype reports of male EL iPSCs showing a normal karyotype (left) and mosaic loss of y chromosome (right). (d) Representative images of teratoma mass hematoxylin and eosin stains from EL iPSC lines representing mesoderm (arrows), endoderm (asterisks), and ectoderm (accent) tissue. Scale bars: 100 μM.

Of the 96 subjects from which PBMCs were collected and isolated, 20 iPSC lines have been generated across EL sub‐groups (Table 2). Subjects chosen for iPSC reprogramming consisted of those at the extremes of health and functionality determined by age group, collected cognitive and ADL score, as well as biological age prediction where applicable. Although 20 subjects were chosen for iPSC reprogramming, this is not exhaustive and more subjects in the bank may be reprogrammed based on experimental needs and specific biological questions and interests. All lines were created using our established methodologies (Park et al., 2017; Somers et al., 2009, 2010, 2012) with at least three independent clones generated from each subject. All lines met stringent quality control parameters for pluripotency and functionality (Park et al., 2017; Somers et al., 2009, 2010, 2012) and were expanded to facilitate sharing in an open‐source approach. Briefly, each line was characterized for undifferentiated ES cell and stemness marker TRA‐1‐81 expression (Figure 2a), for genomic integrity via karyotype analysis (Figure 2b) and comprehensively screened to ensure that created lines were mycoplasma‐free. Although otherwise karyotypically normal, three male centenarians displayed mosaic loss of Y chromosome (Figure 2c). Lastly, teratoma assays were also performed on a subset of lines (6) to confirm pluripotency and the ability to generate tissue types representative of all three germ layers (Müller et al., 2010; Sommer et al., 2009; Takahashi & Yamanaka, 2006) (Figure 2d). Importantly, regardless of subject age, no failures in iPSC generation or pluripotency competence were observed.

**TABLE 2:** Demographic information of EL and non‐EL PBMC and iPSC lines.

Age	Sex	PBMC collected	iPSC generated
100–104	Male	11	1
	Female	17	2
105–109	Male	7	6
	Female	8	5
110+	Male	0	0
	Female	2	2
EL offspring	Male	15	1
	Female	30	3
Non‐EL controls	Male	5	0
	Female	1	0
Total		96	20

*Note*: EL Subjects are classified by age group, sex, and EL status. Included in this table are centenarian offspring as well as non‐EL controls in the offspring age group.

### 2.4 Forward programming of EL‐specific iPSCs into cortical neurons

**FIGURE 3:**
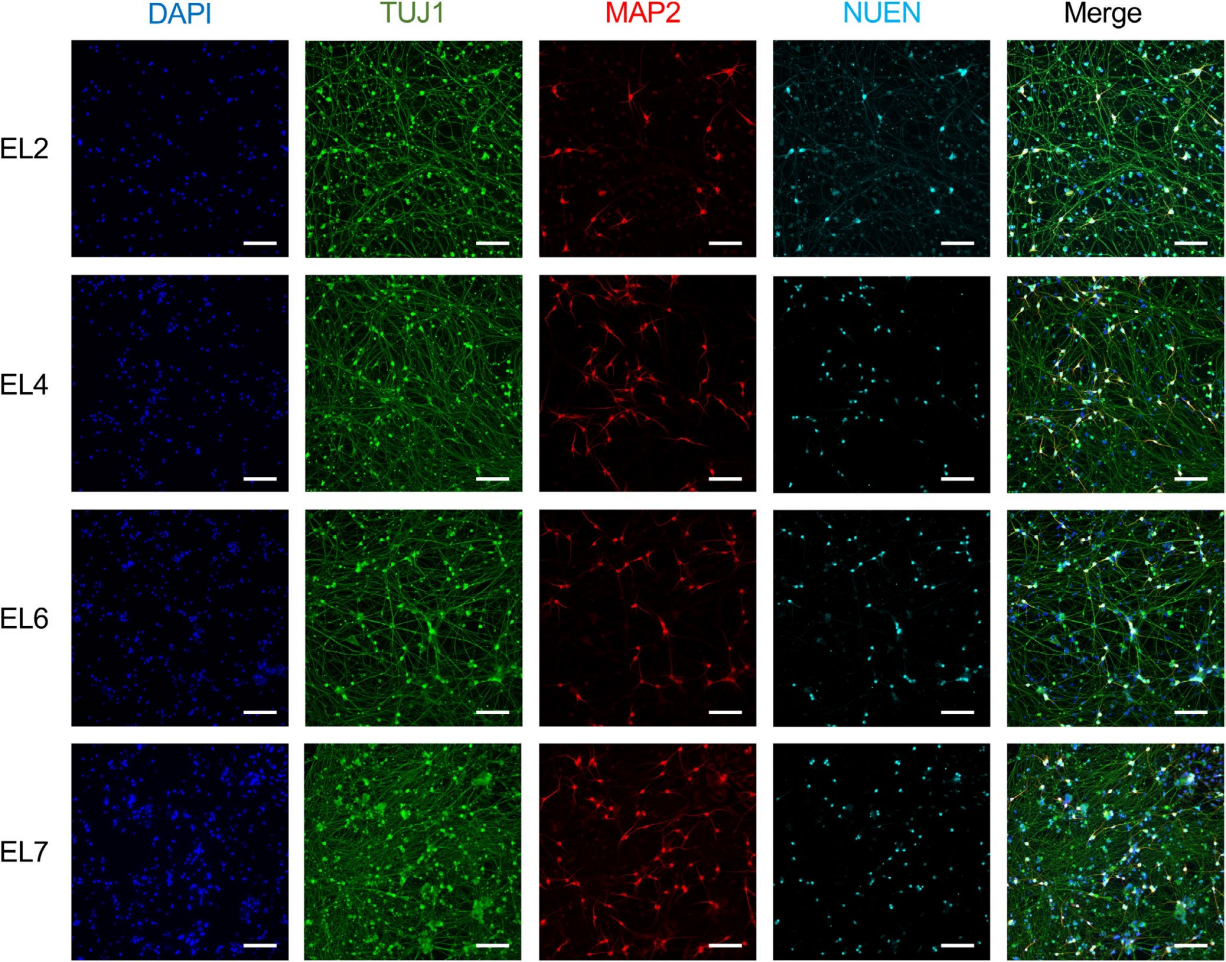
Forward programming of EL‐specific iPSCs into cortical neurons. Representative images from EL iPSC lines brought through forward programming to forebrain cortical neurons. Maturation markers TUJ1 (green), MAP2 (red), NUEN (cyan), and DAPI (blue) are shown. Scale bars: 100 μM.

As a demonstration of the potential of the established EL‐specific iPSC lines to undergo directed differentiation, and as neuronal cell types are impacted by many aging‐related diseases with large socioeconomic burden, we conducted transcription factor‐mediated forward programming to forebrain cortical neurons using established methods (Fernandopulle et al., 2018; Pantazis et al., 2022). Briefly, cells were engineered to have doxycycline‐inducible expression of the neuronal transcription factor neurogenin 2 (NGN2) via transposase‐mediated integration. Upon a 3‐day NGN2 induction using doxycycline, cultures comprised of neural progenitor cells that were replated onto a specialized matrix of laminin and fibronectin and allowed to mature for 14 days in neuronal BrainPhys (STEMCELL Technologies) media supplemented with growth factors NT3, BDNF, and B27. Resulting iPSC‐derived neurons (iNeurons) displayed uniform morphology and expression of neuronal markers TUJ1 (Alexander et al., 1991; Memberg & Hall, 1995), MAP2 (Dehmelt & Halpain, 2004), and NEUN (Gusel'nikova & Korzhevskiy, 2015) (Figure 3). All lines tested performed comparably in their ability to efficiently generate iNeurons.

### 2.5 Subject consent and global distribution of created lines

All of the iPSC lines in this bank were created from subjects using a consent form under the Boston University Institutional Review Board (H32506). This consent form includes a comprehensive template that allows for the unrestricted sharing of deidentified created lines, including potential commercialization and sharing of lines with commercial entities. As a resource to investigators, this consent form has been included as Supplemental Information.

## 3 DISCUSSION

Longevity is multifactorial and complex with a variety of environmental, lifestyle, and genetic determinants (López‐Otín et al., 2023; Sebastiani & Perls, 2012). This last point is highlighted in centenarian offspring, who demonstrate increased resistance to aging‐associated diseases (Adams et al., 2008; Andersen, 2020; Newman et al., 2011; Sebastiani et al., 2009; Terry et al., 2003, 2004) and map younger in aging rate estimators than age‐matched controls (Horvath et al., 2015; Sebastiani et al., 2021). This is consistent in our cohort as judged by both collected phenotypic data and in our application of a series of biological aging clocks. Biological aging clocks have emerged as a prominent tool for measuring and understanding the aging process at a molecular level (Horvath, 2013; Levine et al., 2018; Lu et al., 2019, 2023). Driven by advances in high‐throughput sequencing, computational biology, and machine learning algorithms, researchers are continuously refining existing clocks, developing novel clock architectures, and expanding potential applications. In our study, we observed that many of the centenarians in our cohort demonstrated either significant differences in the comparison of biological to chronological age or slower rates of aging, even though studies have demonstrated that our rate of aging increases over a lifespan (Belsky et al., 2020, 2022). Moreover, genetic variants have been identified which are associated with longevity (Lin et al., 2021; Sebastiani & Perls, 2012; Zhang et al., 2020) and resistance to aging‐related diseases such as AD (Muller‐Gerards et al., 2019; Sebastiani et al., 2019). However, mechanistic insight into how these elements promote longevity remains speculative, highlighting the need for scalable human in vitro models that can be used to understand the gene and regulatory networks that promote resistance to or resilience against aging‐related disease and inform the development of therapeutics which may slow or reverse aging.

As a step toward filling this gap in understanding, we have leveraged access to centenarians and their offspring in the New England Centenarian Study (NECS) (Sebastiani & Perls, 2012), the Longevity Consortium Centenarian Project (LCCP), and Integrative Longevity Omics (ILO) studies to build an EL‐specific library of biomaterial from these exceptionally long‐lived subjects. Using PBMC samples from these individuals, we demonstrated the ability to efficiently generate high‐quality, fully pluripotent iPSCs regardless of subject age. Importantly, we observed no failures in creating iPSCs from these subjects at extreme ages using our standardized reprogramming methodologies (Park et al., 2017; Somers et al., 2010, 2009, 2012). This unique resource provides an unlimited amount of longevity‐specific biomaterial (e.g., genomic DNA) and enables the generation of a multitude of cell and tissue types of aging‐related interest to fuel longevity research and aid in the development and validation of novel geroprotective agents in a human model system. The ability to generate and assay multiple cell types is valuable, as tissue and organ systems have been shown to age at different rates (Nie et al., 2022; Tian et al., 2013). Complementing the molecular profiling performed on the PBMCs of the subjects in our bank, we have curated associated demographics and cognitive and physical function characterizations of the subjects to highlight the relative health and functionality of the participants and their associated iPSC lines. These data should allow for more informed choices of which iPSC lines are best suited for particular research questions or screening applications in aging‐related diseases such as neurodegeneration, where lines derived from those with resiliency or resistance to cognitive decline could serve as valuable models.

Interestingly, during the characterization process, although they were otherwise karyotypically normal, it was noted that a subset of the male centenarian iPSC lines displayed complete loss of the Y chromosome. These findings were confirmed in all clones derived from the subjects. This mosaic loss of Y (mLOY) has been previously identified in the PBMCs of males over 70 and may be a biomarker for aging and susceptibility to and prevalence for aging‐related diseases such as cancer and cardiovascular disease (Kuznetsova et al., 2023; Sano et al., 2022; Thompson et al., 2019). Moreover, and conversely to that seen in centenarians, mLOY is also associated with a significant increase in all‐cause mortality (Thompson et al., 2019). The inclusion of three male centenarians displaying mLOY in our bank allows for interesting research opportunities into the modeling and understanding of this phenomenon. Intriguing possibilities include the use of these particular centenarian lines to identify compensatory mechanisms against the deleterious impact of mLOY or the possibility that mLOY in these individuals is a beneficial adaptation for longevity. Additionally, we demonstrated that iPSC lines in this bank, including those with mLOY, are capable of competent differentiation to produce forebrain cortical neurons. As cortical neurons represent cell types of the central nervous system majorly impacted by the aging process and are intricately involved in aging‐related decline, the ability to generate unlimited numbers of this target cell population from subjects who are potentially resistant to disease should prove to have great utility. Moreover, the inherent flexibility of an iPSC‐based system also allows for the creation of a multitude of aging‐impacted tissue types which can also be used in this capacity.

The use of iPSC‐based platforms to study aging has received skepticism due to the loss of epigenetic information in resulting cells which is a byproduct of the reprogramming process (Mikkelsen et al., 2008). Interestingly, this same process has gained interest in the context of rejuvenation, where the transient expression of the Yamanaka factors may return a cell to a more youthful state without the loss of cell identity, deemed ‘partial reprogramming’ (Macip et al., 2023; Ocampo et al., 2016; Olova et al., 2019). The epigenetic landscape and its associated changes across a lifetime have been identified as a hallmark of aging (López‐Otín et al., 2013, 2023; Yang et al., 2023). However, the genetics of an individual, which strongly impact longevity particularly at extreme ages (Andersen, 2020; Newman et al., 2011; Sebastiani & Perls, 2012), are faithfully captured in iPSCs, enabling the potential discoveries that may arise from this level of information. Additionally, the reset iPSC epigenetic landscape presents a unique opportunity to simulate ‘aging in a dish’ by performing directed differentiations into distinct cell types, thereby reinitiating methylation changes and reinstalling a defined epigenetic landscape.

iPSC‐based systems have revolutionized the modeling of genetic disorders and shown the ability to model diseases that manifest late in life (Ghosh et al., 2023; Giadone et al., 2018, 2020; Kondo et al., 2017; Moreno et al., 2018; Ortiz‐Virumbrales et al., 2017). These platforms allow for a variety of molecular studies to be performed, including those that employ novel gene editing tools to perturb specific genes and pathways associated with longevity. Here, we provide a unique resource of longevity‐specific biomaterial that can be leveraged to build human in vitro models of aging‐related disease and screen potential countermeasures. Models of *human* aging, longevity, and resilience to disease that allow for the functional testing of potential interventions are virtually non‐existent. This resource directly addresses this limitation, while also allowing for cross‐validation of the functional results, identified pathways, and observed signatures across other model systems and laboratories, a major point of concern in the rapidly emerging field of geroscience.

## 4 METHODS

### 4.1 Identification of subjects and curation of clinical history

Centenarians were identified from voter registries, mailings to adult living communities and long‐term care facilities, news articles, and direct participation inquiries to the NECS, LCCP, or ILO studies. Offspring of living or deceased centenarians were also invited to participate. Spouses of the enrolled offspring were invited to participate as a referent group without familial longevity. Centenarian, offspring, and spouse participants complete self‐administered questionnaires to collect sociodemographic, medical history, and physical function data. Participants also complete cognitive screeners and a neuropsychological and physical assessment by video conference or telephone. An informant reported on the presence of cognitive and psychiatric problems in the participant's daily life. Comprehensive phenotypic data are available from the ELITE portal (https://eliteportal.synapse.org/). A convenience sample of NECS, LCCP, and ILO participants was selected for PBMC collection.

Functional independence for centenarians was defined as a score of 80–100 on the Barthel Activities of Daily Living Index (ADLs) (Mahoney & Barthel, 1965), a measure of independence in performing basic self‐care. For the offspring and spouses, functional independence in performing independent living skills such as using a telephone and managing finances, known as IADLs, was defined a score of 14 out of a possible score of 14 on the OARS Multidimensional Functional Assessment Questionnaire (Fillenbaum, 1985). Cognitive status (i.e., cognitively healthy vs. cognitively impaired) was determined by clinical consensus review of neuropsychological assessment scores or, if not available, from cognitive screeners (i.e., the Blessed Information Memory Concentration Test (Blessed et al., 1968) or the Telephone Interview for Cognitive Status (Holtze et al., 2021)).

### 4.2 Collection and isolation of PBMCs

Peripheral blood samples were procured from subjects with EL in the NECS (Sebastiani & Perls, 2012), the LCCP (https://www.longevityconsortium.org/), and the ILO Study (https://longevityomics.org/), including centenarians and their offspring. Additionally, age‐matched spouse controls with no history of EL were collected as controls. In most cases, samples were immediately processed and frozen following isolation of PBMCs via Ficoll gradient. Peripheral blood was drawn and collected into BD Vacutainer™ Glass Mononuclear Cell Preparation (CPT) Tubes (BD 362761). These samples were then centrifuged at 1800  x G for 30 min at room temperature (RT) to separate the blood plasma, PBMC buffy coat, and packed red blood cells. The buffy coat was extracted and washed with phosphate buffer saline (PBS) and centrifuged at 300 × G for 10 min at RT. The cell pellet was resuspended in PBS, counted, and cells were pelleted by centrifugation at 300 × G for 10 min at RT. Cells were resuspended at 8.0 × 10^6^ cells/mL in resuspension buffer and further diluted to 4.0 × 10^6^ cells/mL in freezing medium according to the 10× Genomics protocol (CG00039 Rev D). Cells were then transferred into cryovials and frozen at −80°C before being transferred to long‐term −150°C storage.

### 4.3 Multiparameter flow cytometry characterization of PBMCs

Cytometry characterization was performed according to OMIP‐069 protocol 1 (Park et al., 2020) detailing panel design, validation, and sample staining and acquisition. Frozen PBMCs were thawed rapidly and pelleted via centrifugation. Cells were stained with Live/Dead Fixable Blue dye (ThermoFisher L34961), washed, blocked with Human FcBlock (BioLegend 422301) and stained with a panel of 40 fluorescent reagents (Table S1) supplemented with Monocyte Blocker (BioLegend 426102) and Brilliant Buffer Plus (BD Biosciences 566385). This cell mixture was allowed to stain for 30 min on ice and then washed. Ultracomp beads (ThermoFisher 011‐2222‐42) and control PBMCs were used to include single‐stain controls. Cells and beads were then analyzed on a 5‐laser Aurora spectral flow cytometer (Cytek Biosciences). At least 500,000 cells were recorded for each PBMC sample. Data were processed in SpectoFlo 2.2 (Cytek Biosciences) to generate unmixed features. OMIQ cloud platform was used to perform data cleanup and computational analysis. Live single CD45^+^ single cell datapoints were projected into two‐dimensional space using PCA‐informed opt‐SNE dimensionality reduction algorithm (Belkina et al., 2019) and FlowSOM (Van Gassen et al., 2015) clustering of the same datapoints was overlaid to visualize biological population representation. FlowSOM metaclusters were annotated based on established phenotypic characteristics of the populations.

### 4.4 Targeted methylation arrays and estimation of biological age

Isolation and purification of DNA: PBMCs were thawed rapidly, and DNA was extracted using the Qiagen DNeasy Blood and Tissue Kit (69506). Samples were submitted according to CD Genomics standards for Illumina Infinium MethylationEPIC v2.0 array.

### 4.5 Methylation data quality control and pre‐processing

Quality control metrics for all Infinium MethylationEPIC v2.0 samples were generated through the sesameQC_calcstats function in the SeSAMe R package (v1.20.0) on Bioconductor (v3.18) (Zhou et al., 2018) as detailed in their vignette. Beta values for each sample were obtained from idat files run through the openSesame pipeline from the same package as above, utilizing their recommended preprocessing for human EPICv2 samples. Preprocessing included quality masking for probes of poor design, dye bias correction, detection *p*‐value masking, and background subtraction. Additionally, the EPICv2 array contains some duplicate CpGs across the 900k probes in its microarray, as well as additional suffixes added to probe IDs that reflect design information. To align these identifiers with those from models developed on earlier platforms and facilitate compatibility, the SeSAMe options collapseToPfx = TRUE, and collapseMethod = “mean” were utilized within the beta calling function, in order to remove suffixes, and average the betas from duplicate probes into a single value.

### 4.6 Generating methylation clock estimates

Estimates from several high‐profile age prediction models were performed with the methylation values generated from our samples. The models used were the original Horvath clock (Horvath, 2013), the Hannum clock (Hannum et al., 2013), a centenarian clock (ENCen40+) (Dec et al., 2023), PhenoAge (Levine et al., 2018), and DunedinPACE (Belsky et al., 2022). The Illumina EPICv2 platform comprises a new set of CpGs, which largely overlaps those found in EPICv1 and earlier platforms, but not entirely. As a result, each of these models had a small number of CpGs whose values were imputed to generate estimates as accurately as possible. For each model, imputation was followed as detailed in the corresponding manuscripts, if available. Otherwise, mean beta values were imputed from an external dataset belonging to the same Illumina platform on which a model was trained. For the (Horvath, 2013) model, approximately 13 of the 353 CpGs (3.68%) were missing from EPICv2, and missing values were obtained using mean DNAm values from a gold standard dataset detailed in the original supplement S2 (materials and methods) of the manuscript. Imputation with the centenarian clock (ENCen40+), as well as the PhenoAge clock, was done using mean DNAm values of the controls from a peripheral blood EPICv1 dataset GSE157252 (Piao et al., 2022) to supplement missing values for 39 of the 559 CpGs (6.98%) used by ENCen40+ and the 18 of 513 (3.51%) used by PhenoAge respectively. For the Hannum model, 7 CpGs of the 71 were missing (9.86%), and imputation was performed using the GSE40279 dataset (Illumina 450k) used in his original manuscript. Lastly, predictions for the DunedinPACE clock were generated using the Github repository (https://github.com/danbelsky/DunedinPACE) maintained by the authors. This repository has been independently updated to work with EPICv2 data, as the authors note that 29 of the 173 CpGs (16.76%) from the original model no longer appear in the new methylation array. The authors of this model utilize a similar approach of substitution of averages from Dunedin beta values for correction of missing data from their model, as well as for a panel of 19,827 probes for background normalization in the calculations.

### 4.7 iPSC creation, expansion, and distribution

Previously isolated and frozen PBMCs were thawed rapidly and erythroblasts were expanded using erythroblast expansion medium (EM) for 9 days. A comprehensive methodology concerning peripheral blood collection, PBMC isolation, expansion, and our approach to reprogramming can be found in Sommer et al., 2012 with adaptations including the use of sendai virus‐mediated reprogramming factor delivery as well as a feeder‐free protocol. Briefly, following erythroblast expansion cells were counted and 2.0 × 10^5^ cells were transduced using the Invitrogen™ CytoTune™‐iPS 2.0 Sendai Reprogramming Kit (A16517). The next day, cells were collected and transferred onto matrigel‐coated wells. Over the first 5 days post‐transduction, the cells were slowly transferred to ReproTeSR (STEMCELL Technologies 05926) by adding the ReproTeSR on Days 3 and 5 without aspirating the existing EM. From Day 5 onward, the cells were cultured fully in ReproTesR. Once colonies adhered and were large enough, a cross‐hatching method was used to divide each colony and passage onto a Matrigel‐coated well to establish a clonal culture. These clones were expanded to at least 30 early passage vials and stored for sharing with the longevity community. All generated iPSC lines passed stringent quality control and functional standards for pluripotency and directed differentiation including those established by our NIH U01 HL07443 quality control working group.

### 4.8 Teratoma assay

Three female NU/NU immunocompromised mice (Jackson Labs strain#002019) per subject were injected subcutaneously in the flank with 1 × 10^6^ iPSCs (controlled for passage number) from each respective iPSC line suspended in high content Matrigel (Corning™ 354263) supplemented with 10 μM Y27632 (Reprocell). Mice were monitored for teratoma formation for up to 20 weeks. Following teratoma development, the mass was resected, fixed in 4% PFA at RT overnight, and paraffin embedded and sectioned for hematoxylin and eosin staining.

### 4.9 Forward programming of iPSCs to cortical neurons

Neurogenin 2 (NGN2) was expressed in iPSC lines using a tetracycline inducible promoter (Addgene 172115) integrated using Piggybac plasmid EFa1‐Transposase (Addgene plasmid 172116) via lipofectamine (ThermoFisher STEM00015) as described previously (Pantazis et al., 2022). Selection was performed after 48 h using 2 μg/mL puromycin for 14 days. Stably integrated lines were dissociated with accutase (ThermoFisher A1110501) and plated at single‐cell onto matrigel coated plates with induction media containing 10 μM Y‐27632 and 2 μg/mL doxycycline (Sigma D9891) as previously described (Fernandopulle et al., 2018). Following 3 days in induction medium containing doxycycline, cells were dissociated using accutase (ThermoFisher A1110501) and plated onto poly‐L‐ornithine (Sigma P4957), poly‐D‐lysine (Gibco A3890401), 10 μg/mL Fibronectin (Corning 356008), and 10 μg/mL Laminin (Gibco 23017015) coated plates in cortical neuron culture medium (CM) containing B27 (Gibco 17504044), BDNF (R&D Technologies 248‐BDB), and NT3 (R&D Technologies 267‐N3) (Fernandopulle et al., 2018). Cells were patterned for 14 days in this medium before being used for downstream assays.

### 4.10 Immunofluorescence

Cells were washed with PBS and fixed using 4% paraformaldehyde (Electron Microscopy Sciences 15714S) for 20 min at RT. Cells were permeabilized and blocked using 5% Fetal bovine serum (ThermoFisher 16141079), 0.3% Triton X‐100 (Sigma T8787), and 2% bovine serum albumin (Gibco 15260037) diluted in PBS for 1 h at RT. Cells were stained with primary antibodies (Table S2) diluted in block/perm buffer overnight at 4°C. Cells were then washed three times with block/perm buffer and stained with corresponding secondary antibodies for 30 min at RT. Cells were washed three times with block/perm buffer and stained with Dapi nuclear stain (ThermoFisher 62248) diluted in fix/perm buffer for 10 min at RT. Cells were washed three times with block/perm buffer and imaged.

## AUTHOR CONTRIBUTIONS

*Conceptualization*: T.W.D., G.J.M., T.P., P.S., and S.A. *Methodology*: T.W.D., G.J.M, T.P., S.A. *Investigation*: T.W.D., G.J.M., S.F.C, N.S., Y.L., R.M.G, A.C.B. *Writing–Original draft*: T.W.D., G.J.M., T.P., P.S., S.A., N.S. *Funding Acquisition*: G.J.M., T.P., P.S., S.A. *Resources*: B.G., B.P., D.V., J.S., S.M., M.F.J. *Data Curation*: M.D., S.A.

## CONFLICT OF INTEREST STATEMENT

The authors declare no competing interests.

## Supporting information


Data S1.

## Data Availability

The data that support the findings of this study are openly available in the ELITE portal at https://eliteportal.synapse.org/ and has been deposited under GEO accession number GSE276957.
