# Supplementary material for: A longevity‐specific bank of induced pluripotent stem cells from centenarians and their offspring
Source: Aging Cell. 2024 Sep 25;24(1):e14351. doi: 10.1111/acel.14351 (PMC11709102; doi:10.1111/acel.14351)
Supplement: Supplementary file 1 — Data S1. [file ACEL-24-e14351-s001.pdf]

Supplementary Figure 1. Multi-dimensional flow cytometric characterization of the PBMCs of individuals with exceptional longevity.

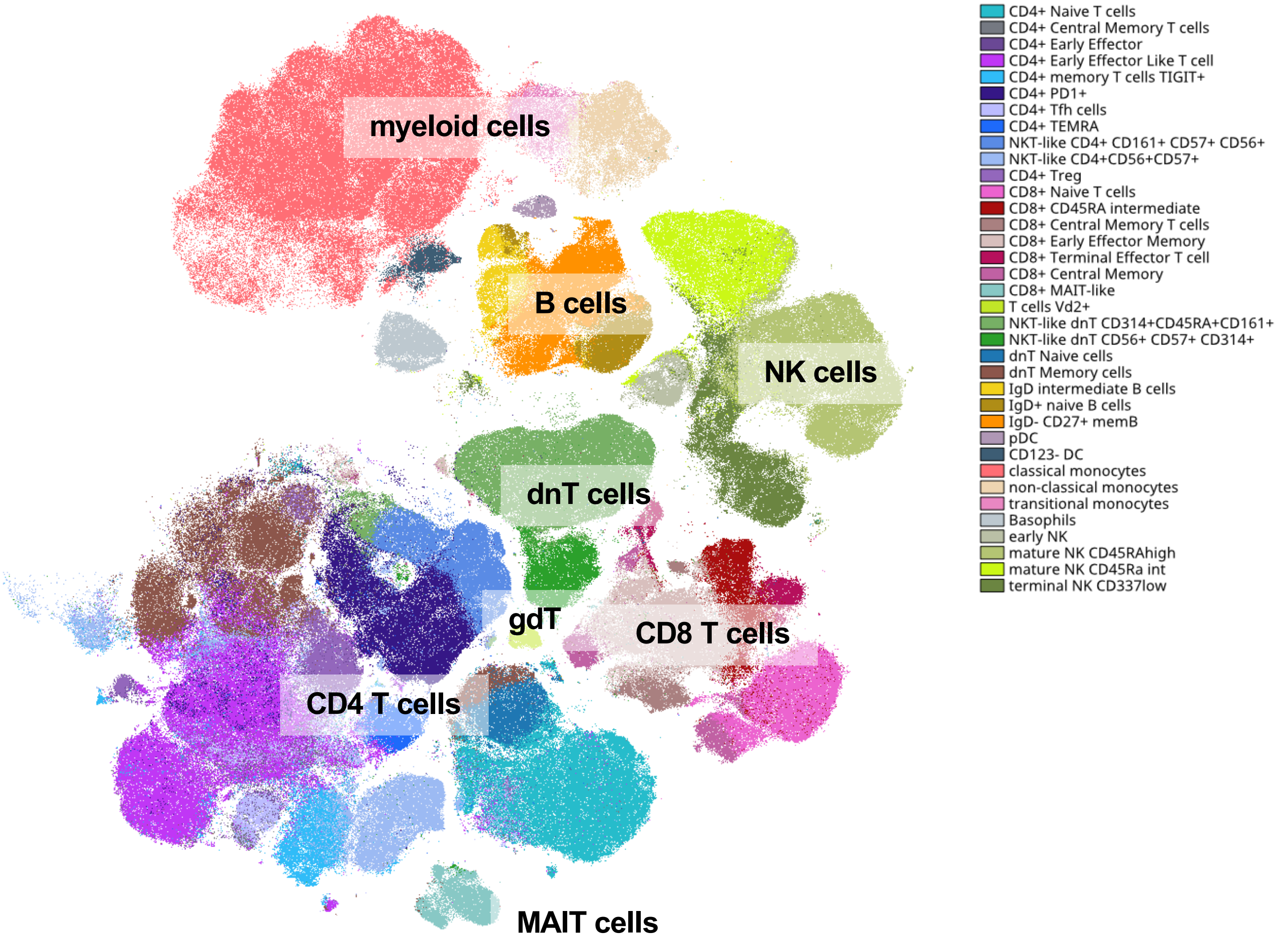

Supplementary Figure 1. Multi-dimensional flow cytometric characterization of the PBMCs of individuals with exceptional longevity. PCA-informed opt-SNE visualization of 40-color cytometry-characterized PBMCs with FlowSOM[82] clustering overlayed to annotate clusters based on established phenotype characteristics.

Supplementary Figure 2. Phenotypic identification of PBMC sub-types within individuals with exceptional longevity.

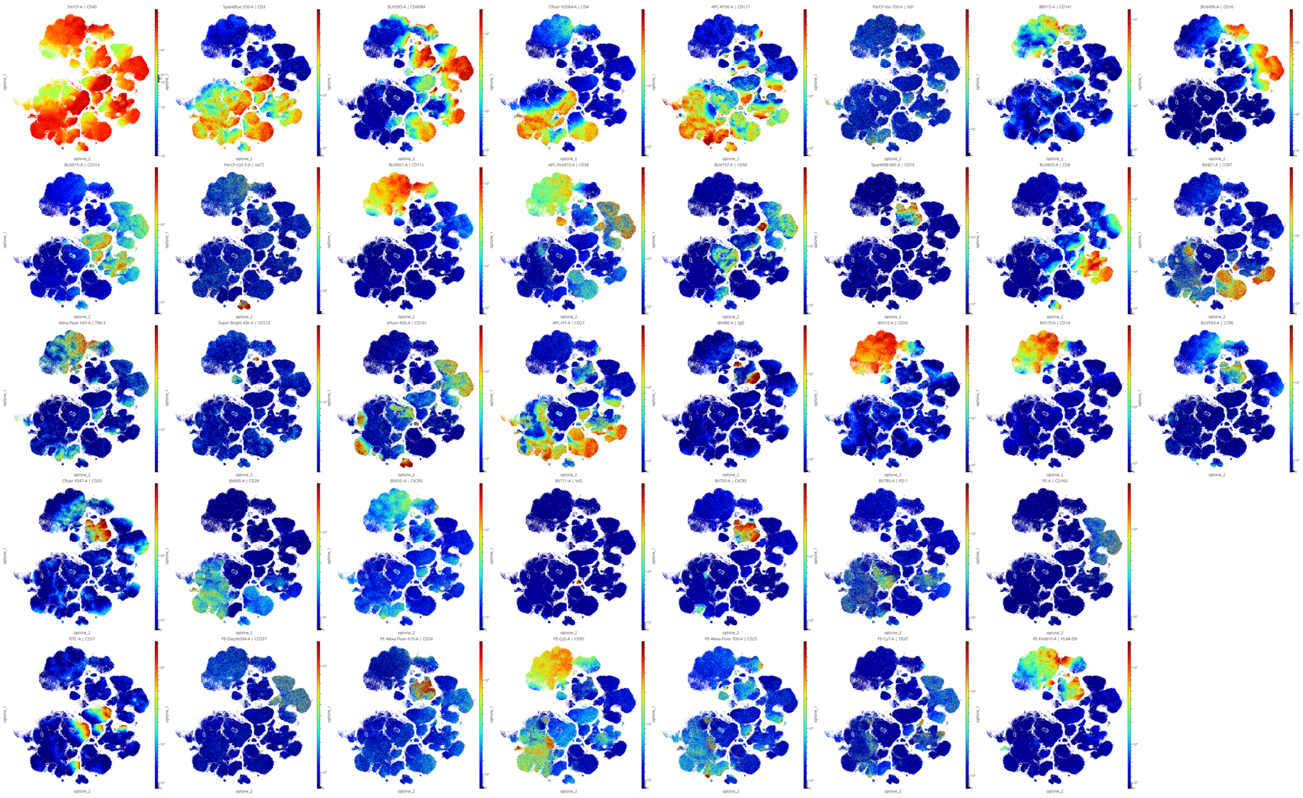

Supplementary Figure 2. Phenotypic identification of PBMC sub-types within individuals with exceptional longevity. Individual marker expression pattern overlayed on opt-SNE visualization of PBMCs based on established phenotypic characteristics.

Supplementary Table 1. List of antibodies used for multiparameter flow cytometry characterization of PBMCs .

Ag	Fluor	Vendor	Cat No	amount per 1 test, ul
CD45RA	BUV395	BD Biosciences	740298	1.2
CD16	BUV496	BD Biosciences	612944	0.6
CCR6	BUV563	BD Biosciences	749362	3
CD314	BUV615	BD Biosciences	751232	5
CD11c	BUV661	BD Biosciences	612967	1.2
CD56	BUV737	BD Biosciences	564447	1.2
CD8	BUV805	BD Biosciences	612889	1.2
CCR7	BV421	BD Biosciences	740052	5
CD123	SB436	Thermo Fisher	62-1239-42	2.5
CD161	eFluor 450	Thermo Fisher	48-1614-42	5
IgD	BV480	BD Biosciences	566138	0.6
CD33	BV510	Biolegend	303422	2.5
CD20	PacOrange	Thermo Fisher	MHCD2030	5
CD14	BV570	Biolegend	301831	3
CD28	BV605	Biolegend	302968	2.5
CXCR3	BV650	Biolegend	353730	2.5
Vd2	BV711	Biolegend	331412	5
CXCR5	BV750	BD Biosciences	747111	1.2*
PD-1	BV785	Biolegend	329930	5
CD141	BB515	BD Biosciences	565084	2.5
CD57	FITC	Biolegend	359604	1.2
CD3	SparkBlue550	Biolegend	344852	2.5
CD45	PerCP	Biolegend	304025	1.2
Valpha7.2	PerCPCy5.5	Biolegend	351709	2.5
Vd1	PCPVio700	Miltenyi	130-120-581	1
CD160	PE	Thermo Fisher	12-1609-42	5*
CD4	YG584	Cytek Biosciences	R7-20042	1.2
CD337	PEDz594	BD Biosciences	325231	2.2
CD24	PEAF610	Thermo Fisher	MHCD2422	2.5
CD95	PECy5	Biolegend	305610	0.6
CD25	PEAF700	Thermo Fisher	MHCD2524	5
TIGIT	PECy7	Biolegend	372714	1.2
HLADR	PEFire810	Biolegend	900000155	5
CD1d tetramer	APC	NIH tetramer core		0.02
TIM-3	AF647	BD Biosciences	565558	2.5
CD19	SparkNIR685	Biolegend	302269	1.2
CD127	APCR700	BD Biosciences	565185	6
CD27	APCH7	BD Biosciences	560222	2.5
CD38	APCFire810	Biolegend	303550	3.2

* reagents added to the cells before the rest of the reaction mix

Supplementary Table 2. List of antibodies used for immunofluorescence.

Antibody	Vendor	Cat No	Dilution
Tra-1-81	Thermo Scientific	MA1024	1:100
Dapi	Invitrogen	D1306	1:1000
Tuj1	Millipore Sigma	T2200	1:200
Map2	Abcam	ab5392	1:1000
NeuN	Thermofisher	66836-1-IG	1:150
